# Predicted Structure and Functions of the Prototypic Alphaherpesvirus Herpes Simplex Virus Type-1 UL37 Tegument Protein

**DOI:** 10.3390/v14102189

**Published:** 2022-10-04

**Authors:** Therese Marie A. Collantes, Carolyn M. Clark, Farhana Musarrat, Nithya Jambunathan, Seetharama Jois, Konstantin G. Kousoulas

**Affiliations:** 1 Division of Biotechnology and Molecular Medicine and Department of Pathobiological Sciences, School of Veterinary Medicine, Louisiana State University, Baton Rouge, LA 70803, USA; 2 College of Veterinary Medicine, University of the Philippines Los Baños, Los Baños, Laguna 4031, Philippines; 3 School of Medicine, Indiana University, Indianapolis, ID 46202, USA; 4 School of Basic Pharmaceutical and Toxicological Sciences, College of Pharmacy, University of Louisiana Monroe, Monroe, LA 71201, USA

**Keywords:** computational modelling, molecular dynamics simulation, functional modelling, web-based, AlphaFold, herpesvirus, tegument protein, UL37, retrograde transport

## Abstract

The alphaherpesvirus UL37 tegument protein is a highly conserved, multi-functional protein. Mutagenesis analysis delineated the UL37 domains necessary for retrograde transport and viral replication. Specifically, the amino-terminal 480 amino acids are dispensable for virus replication in epithelial cell culture, but it is unknown whether this amino-terminal deletion affects UL37 structure and intracellular transport in epithelial cells and neurons. To investigate the structure and function of UL37, we utilized multiple computational approaches to predict and characterize the secondary and tertiary structure and other functional features. The structure of HSV-1 UL37 and Δ481N were deduced using publicly available predictive algorithms. The predicted model of HSV-1 UL37 is a stable, multi-functional, globular monomer, rich in alpha helices, with unfolded regions within the linker and the C-tail domains. The highly flexible C-tail contains predicted binding sites to the dynein intermediate chain, as well as DNA and RNA. Predicted interactions with the cytoplasmic surface of the lipid membrane suggest UL37 is a peripheral membrane protein. The Δ481N truncation did not alter the predicted structure of the UL37 C-terminus protein and its predicted interaction with dynein. We validated these models by examining the replication kinetics and transport of the Δ481N virus toward the nuclei of infected epithelial and neuronal cells. The Δ481N virus had substantial defects in virus spread; however, it exhibited no apparent defects in virus entry and intracellular transport. Using computational analyses, we identified several key features of UL37, particularly the flexible unstructured tail; we then demonstrated that the UL37 C-terminus alone is sufficient to effectively transport the virus towards the nucleus of infected epithelial and neuronal cells.

## 1. Introduction

Herpesviruses belonging to the subfamily *Alphaherpesvirinae* are an important group of human and animal pathogens that cause rapid lytic replication at the site of infection and primarily establish latent infection in the sensory ganglia of the host. Some of the most important representatives of subfamily *Alphaherpesvirinae* that affect humans include human simplex virus-type 1 (HSV-1), HSV-2 and varicella-zoster virus (VZV). Among alphaherpesviruses found in animals, pseudorabies virus (PrV), bovine herpesvirus 1 (BoHV-1) and gallid herpesvirus 2 (GaHV-2/Marek’s disease virus) are of significant veterinary and economic importance [1,2]. Vaccination against herpesviruses in veterinary settings is routinely performed using live attenuated viruses in livestock and small animals [3,4,5]. However, in humans, safe and efficacious preventive or therapeutic vaccines against HSV-1 have yet to be approved by the FDA. The novel basis of rational vaccine design largely depends on current knowledge of determinants of viral pathogenesis. One approach to developing live-attenuated vaccines against herpesviruses is introducing mutations on highly conserved viral genes with functions in immune evasion [6]. The goal is to achieve immunogenicity without diminishing viral replication. Thus, understanding the structure and function of virus-encoded proteins, especially those used by the virus to evade the host immune system, can lay the groundwork for further vaccine studies.

Herpesvirus virions appear similar in morphology across subfamilies, consisting of a double-stranded DNA genome and more than 30 viral capsid, tegument, and envelope proteins. A portion of these structural proteins is found in the tegument, a dense matrix of proteins between the lipid envelope and nucleocapsid of herpesviruses. In HSV-1, 23 virally encoded tegument proteins have been identified by mass spectrometry [7]. Tegument proteins play a key structural role in viral maturation and assembly, where the addition of tegument to the viral capsid within the nucleus and trans-Golgi network (TGN) appears to be ordered and elaborate [8]. Moreover, tegument proteins facilitate viral replication by regulating the host immune response. By interacting with pathogen recognition receptors (PRRs), tegument proteins can suppress multiple innate immune signaling pathways, thereby promoting viral replication [9]. Interactions between tegument proteins and cellular targets appear to be highly complex, with a multitude of contact points between the viral and cellular proteins occurring at each viral replication step [8,9]. Identification of viral and cellular protein interactions has increasingly been reported; however, with recent advances in predictive modeling technology, a comprehensive map of potential interactions can be developed using a computational approach.

Tegument proteins are divided into inner and outer layers depending on their capsid association following cell entry. Outer tegument proteins become detached from the capsid, while inner tegument proteins remain bound to the capsid [10,11,12]. UL37 is a 120 kDa protein within the inner tegument layer of herpesviruses. Along with UL36 and US3, UL37 continues to associate with the capsid following cellular entry and is involved in intracellular capsid transport [10,11,12].

### 1.1. UL37 Functional Domains

**Figure 1:**
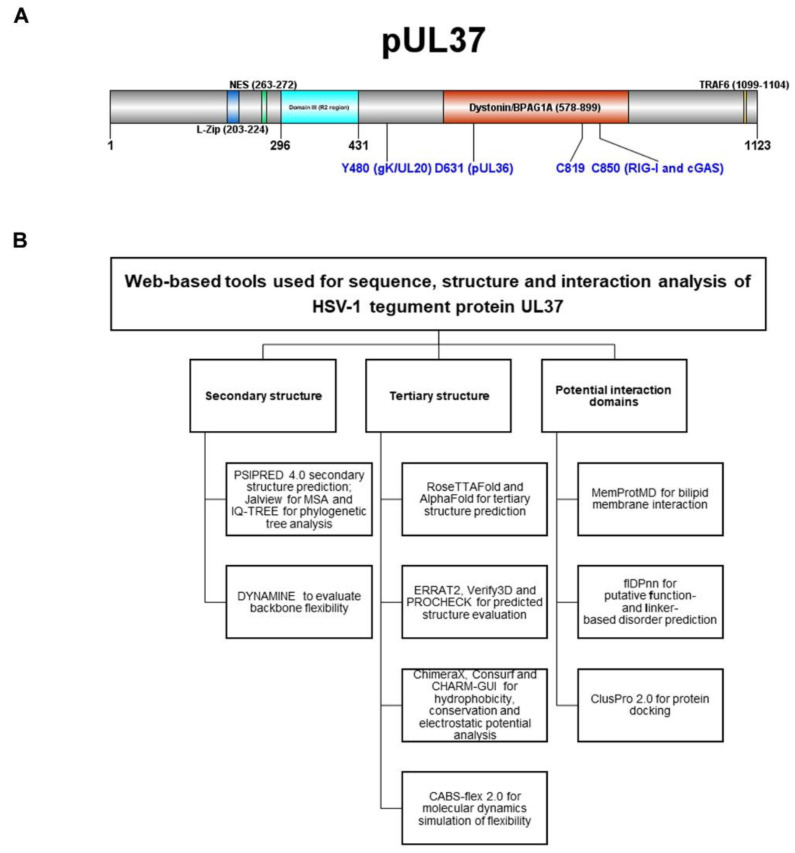
(**A**) Schematic diagram of HSV-1 UL37 tegument protein functional domains. Amino-and carboxy-termini show previously identified functional domains for viral replication and innate immune regulation. (**B**) Multiple web-based computational tools and software used for sequence predicted structure and interaction analysis of tegument protein UL37. Webservers and software are grouped according to secondary and tertiary structure and interaction domain analysis.

Primary and alternate functions of UL37 in virion morphogenesis, capsid transport and innate immune regulation have been described. A schematic diagram of HSV-1 UL37 tegument protein shows several identified functional domains in the amino and carboxy-termini (Figure 1A). A leucine zipper (L-zip) motif (aa203–224) and nuclear export signal (aa263–272) are found in the amino terminus of UL37 [13,14]. The R2 region in domain III of the amino terminus of UL37 (aa296–431) is critical for retrograde axonal transport within neurons [15]. A highly conserved tyrosine residue in position 480 (Y480) of UL37 plays a key role in interaction with membrane protein gK for cytoplasmic virion envelopment and viral replication [16]. As a tegument protein associated with the major tegument protein UL36, UL37 is essential to virus maturation, specifically for outer tegumentation and secondary envelopment [17]. UL37 is involved in retrograde cellular transport. Amino acids 578–899 of the carboxy terminus of UL37 are the interacting domain of dystonin (BPAG1), an important non-motor protein involved in bridging cytoskeleton networks for retrograde and anterograde transport in epithelial, muscular, and neuronal cells [18]. UL37, together with Vp5, interacts with the dynein intermediate chain (DIC) as a mechanism for capsid transport to the nucleus [19]. As a regulator of host innate immune response, UL37 functions as a pseudoenzyme, with two cysteine residues (C819 and C850) facilitating a post-translational modification called deamidation on pathogen recognition receptors (PRRs), cGAS and RIG-I [20,21,22]. The tail end of the UL37 C-terminus (aa1099–1104) contains a TRAF6-binding domain that activates NFƙB signaling.

Functional domains of HSV-1 UL37 have been previously investigated through extensive mutagenesis analyses; however, it remains unclear how UL37 functions in retrograde transport. HSV-1 tegument protein UL37, together with Vp5, interacts with the dynein intermediate chain (DIC) as a mechanism for capsid transport to the nucleus; however, the precise interaction domain has yet to be elucidated [19]. Deletion of the C-terminal 133 amino acids of UL37 is lethal to the virus [23]. In contrast, the first 480 amino-terminal amino acids are dispensable for virus growth, although they inhibit virus spread.

### 1.2. Computational Modeling of the HSV-1 UL37 Protein

Predictive modeling is used to gain insight into protein structure and facilitate experimental structural studies [24]. Recently, significant improvements were made to the prediction of protein structure using neural network-based approaches [25]. AlphaFold and RoseTTAFold utilize machine learning approaches to predict highly accurate protein structures based on millions of parameters learned from training datasets of known experimental protein structures [26]. With its innovative machine learning approach to protein structure prediction, AlphaFold has built the AlphaFold Protein Structure Database, which contains proteome-wide predictions for 16 species, including humans, fruit flies, *E. coli* and maize, available within the AlphaFold Protein Structure Database [25,27]. Because of the severe acute respiratory syndrome coronavirus 2 (SARS-CoV-2) pandemic, AlphaFold has been applied to model the viral proteome of SARS-CoV-2, including those structures of understudied proteins which have not yet been experimentally determined, with considerable success [28]. In recent biannual meetings of Critical Assessment of Structure (CASP14), RoseTTAFold placed second among all groups, including the Zhang server and IntFold6. RoseTTAFold uses a three-track neural architecture to attain a competitive average TM-score of prediction methods compared to AlphaFold [29]. By simultaneous processing of amino acid sequence (1D), distance (2D) and coordinate (3D) data, RoseTTAFold offers another novel approach to solving the protein structure problem.

Because most of the resolved x-ray crystal structures of tegument proteins are partial, there is limited experimental data available to predict protein structures using template-based modeling (TBM) [30]. Therefore, applying deep learning-based methods to predict the tegument structures is warranted. The main advantage of deep learning to predict difficult-to-resolve proteins is the ability to generate a high-quality model even with limited experimental data [26]. In this paper, we utilize a combination of readily available computational tools for the characterization and interaction analysis of the predicted structure of tegument protein UL37. Deletion of the amino-terminal 480 amino acids of the HSV-1 UL37 protein did not appreciably affect the predicted structure of the remaining portion of UL37. We validated these models by showing that the deletion of the amino terminal 480 amino acids of the HSV-1 UL37 protein did not affect the ability of the mutated virus to infect and efficiently transport toward the nucleus of infected cells, including neurons.

## 2. Materials and Methods

We used a three-way strategy to select and apply multiple computational tools to characterize the predicted structure of tegument protein UL37 (Figure 1B). Active links to web-based tools and software are listed in Supplementary Table S1.

### 2.1. Server and Software Input

The amino acid sequence of the HSV-1 (McKrae) UL37 protein was retrieved from GenBank (Accession Number: AFP86401.1) and used as input for secondary and tertiary structure prediction.

### 2.2. Phylogenetic Analysis, Generation of Predicted Secondary and Tertiary Models for UL37 and Evaluation of Model Quality

A phylogenetic tree of *alphaherpesviruses* was constructed using IQ-TREE under maximum likelihood with ultrafast bootstrapping [31,32,33]. Input sequences used for multiple sequence alignment (MSA) in Jalview were selected from a standard protein BLAST search and are listed in Supplementary Table S2 [34]. An orthologue of UL37 from human herpesvirus-5 (human cytomegalovirus), tegument protein UL47 (YP_081505.1), was used as an outgroup for rooting the tree. The predicted secondary structure was generated using PSIPRED 4.0 [35]. To predict the tertiary structural model, we selected two highly accurate methods, AlphaFold2 (Google Colaboratory) and RoseTTAFold, which both employ neural networks for protein prediction [25,27,29]. ERRAT2 [36], PROCHECK [37], and VERIFY3D [38,39] were used to evaluate and compare the quality of UL37 predicted models. For comparison among alphaherpesviruses, structural models of bovine herpesvirus 1 (BoHV-1) and pseudorabies virus (PrV) were predicted using AlphaFold2 with NCBI reference sequence AFV53381.1 and YP_068340.1 as sequence input, respectively.

### 2.3. Identification of Disordered and Binding Regions

Disordered and binding regions were identified and evaluated using DISOPRED3 and DynaMine [40,41]. We determined DNA, RNA and protein-binding sites within disordered regions using the Putative Function-and Linker-based Disorder Prediction using a deep neural network webserver (fldPnn) [42].

### 2.4. Molecular Dynamic Simulation of Structural Flexibility

To characterize the structural flexibility of UL37, CABS-flex 2.0 was used to conduct a coarse-grained molecular dynamics (CGMD) simulation [43]. Simulation parameters used were as previously described, where coarse-grained simulations were derived from all-atom simulations of 10 nanosecond lengths [43,44]. Global C-alpha and side chain restraints weight were set to 1.0. To test the effect of temperature and identify flexible regions susceptible to unfolding, three settings were tested at 50 cycles each; T = 1.0 (near crystal temperature), T = 1.40 and T = 2.0 (temperature allowing unrestrained protein chains to unfold fully). Root mean square fluctuation (RMSF) analysis was performed to compare changes in the flexibility of amino acid residues at increasing temperatures.

### 2.5. Hydrophobicity, Evolutionary Conservation, and Electrostatic Surface Potential

The surface characteristics of UL37 were visualized and analyzed using the following approaches. Hydrophobic and hydrophilic residues were visualized using UCSF ChimeraX. ConSurf was used to visualize evolutionary conservation [45]. PBEQ solver generated electrostatic surface potential maps in CHARMM-GUI [46,47].

### 2.6. Molecular Dynamic Simulation of Lipid Membrane Interaction

MemProtMD was used to determine UL37 interaction with a lipid bilayer membrane. A Google Colab tool was made available to run MemProtMD with the predicted model of UL37 as input. Briefly, MemProtMD uses MEMEMBED to determine the structural orientation of the protein relative to a lipid membrane. Initially, the size of the box is 80 Å at the *z*-axis. In the x and y direction, the distance between the protein and the end of the axis is at least 30 Å. Dipalmitoylphosphatidylcholine (DPPC) lipid molecules were used. Specifically, the bilipid membrane type selected was built using 1-palmitoyl-2-oleoyl-sn-glycero-3-phosphoethanolamine (POPE), 1-palmitoyl-2-oleoyl-sn-glycero-3-phosphoglycerol (POPG) and cardiolipin (CL). Within Google Colab, the coarse-grained molecular dynamics (CGMD) simulation was performed for a total duration of 60 ns in length. Using the MARTINI 2.1 forcefield, the lipid bilayer membrane was built. At the end of the 60 ns simulation, a snapshot was taken and converted from coarse-grained resolution to atomic resolution using the CG2AT-align method [48].

### 2.7. Protein Docking

We utilized ClusPro 2.0 to perform protein docking of the predicted model of UL37 to dynein [49,50]. Two dynein structures were tested-dynein light chain-intermediate chain (LC-IC) complex from *Drosophila* sp. (PDB ID 2PG1); and the predicted structure of the human cytoplasmic dynein 1 intermediate chain 1 (AlphaFoldDB Identifier: AF-O14576-F1). The dynein LC-IC complex PDB file was retrieved from the RCSB Protein Data Bank. The predicted structure of human cytoplasmic dynein 1 intermediate chain 1 was retrieved from the AlphaFold Protein Structure Database.

### 2.8. Cell Lines and Viruses

African green monkey kidney cells (Vero) and SH-SY5Y were obtained from the American Tissue-Culture Collection (ATCC) (Rockville, MD, USA). Veros were maintained on Dulbecco’s Modified Eagle Medium (Gibco-BRL, Grand Island, NY, USA) supplemented with 10% fetal bovine serum (Gibco-BRL, Grand Island, NY, USA) and 100 ug/mL Primocin© (Invitrogen, INC., Carlsbad, CA, USA). SH-SY5Y were maintained on Eagle’s Minimum Essential Medium (ATCC, Rockville, MD, USA) supplemented with 15% fetal bovine serum (Gibco-BRL, Grand Island, NY, USA) and 100 ug/mL Primocin© (Invitrogen, INC., Carlsbad, CA, USA). The wildtype (WT) and Δ481N viruses were a gift from Prashant Desai (Johns Hopkins University, Baltimore, MD, USA). The mutants were made on a KOS background with a GFP on UL37. Viruses were grown and titrated on Vero cells [23].

### 2.9. Growth Analysis of WT and Δ481N Mutant Virus

Growth kinetics were performed by infecting confluent monolayers of Vero with WT or Δ481N at an MOI of 1.0 for 0, 6, 12, 24, and 48 h post-infection (hpi). Plates were frozen and thawed thrice, cell lysates collected, and titrated on Vero. Experiments were performed in triplicate. Plaque assay was performed using a 1% methylcellulose gel overlay on Vero cells infected with either WT or Δ481N mutant virus. After 96 h of incubation, cells were fixed with cold methanol and placed at −20 °C for 20 min before staining using immunohistochemistry. The primary antibody was a rabbit polyclonal anti-herpes simplex virus type 1 antibody (Dako Denmark A/S, Glostrup, Denmark). Vector© NovaRED peroxidase substrate kit was used for colorization.

### 2.10. Intracellular Transport Assay in Epithelial and Neuronal Cell Lines

Vero cells were grown overnight in 8-well chamber slides. Cells were adsorbed at 4 °C with either wildtype or Δ481N mutant virus at MOI = 50 for 1 h. After 1 h, the cells were washed with low pH buffer to remove the unbound viruses. Then, warm media was added, and cells were placed at 37 °C for 10 min, 1 h and 6 h incubation. Following incubation, cells were fixed and permeabilized with cold methanol for immunofluorescence staining. Primary antibodies against rabbit α-tubulin (Abcam, Inc., Cambridge, MA, USA) and mouse anti-HSV-1 ICP5 (Santa Cruz Biotechnology, Dallas, TX, USA) were used to visualize viral capsid transport. Secondary antibodies used were anti-rabbit Alexa Fluor^TM^ 488 and anti-mouse Alexa Fluor^TM^ 647. Nuclei were stained with DAPI (49,6-diamidino-2-phenylindole). Images were taken using a 63x objective (oil immersion) on a fluorescence microscope (Zeiss Axio Observer Z1), with Zen software for image processing.

SH-SY5Y cells were grown and differentiated into neurons, as previously described [51]. Fully differentiated SH-SY5Y cells were seeded into 150μm standard neuron devices (Xona Microfluidics, Research Triangle Park, NC, USA) with differentiation media #3 onto lysine-coated plates as described [51]. Matrigel Basement Membrane Matrix (Corning Inc., Corning, NY, USA) was added to the top of wells containing neuronal cell bodies. Cells were monitored for axon growth through the channels of the microfluidic device for up to 2 weeks, with media change every other day. When axons were ready for infection, media was removed from the axonal side of the device, and Veros were seeded overnight. Then, medium from the axonal/Vero side was removed again, and 200,000 PFU of either HSV-1 WT or Δ481N were added to the wells. Cells were incubated at 37 °C for 12 hpi until images were taken on a fluorescent microscope (Zoe Fluorescent Cell Imager).

## 3. Results

### 3.1. Phylogenetic Analysis of Alphaherpesviruses Based on Tegument Protein UL37 Orthologs

We derived the phylogenetic tree using a set of alphaherpesvirus UL37 orthologs determined using a standard protein BLAST search with AFP86401.1 as the query sequence within Alphaherpesvirinae (taxid 10293) organisms and Reference Proteins (refseq_protein) as database. We analyzed the extent to which amino acid sequences of alphaherpesvirus UL37 orthologs were similar to HSV-1 UL37 by examining percent identity (% identity), tabulated in the standard protein BLAST search result (Supplementary Table S2). We found that within the genus simplex virus, % identity ranged from 45.62% to 84.68%. Human alphaherpesvirus 2 (84.68%), Macacine alphaherpesvirus 1 (71.85%), Papiine alphaherpesvirus 2 (71.40%) and Cercopithecine alphaherpesvirus 2 (71.03%) showed the highest similarity to HSV-1. A significantly lower range of % identity was found among members of the genus varicellovirus (27.65% to 31.68%) and genus mardivirus (26.71% to 28.37%).

We sought to identify notable differences between the amino acid sequence of the HSV-1 (McKrae) UL37 protein and alphaherpesvirus orthologs by using Clustal Omega multiple sequence alignment (MSA) in Jalview (Supplementary Figure S1). The first 25 amino acids of the HSV-1 UL37 protein have conservation scores of 0–1 compared to representatives of alphaherpesviruses. The central region contained sequences (ATPLSALLP) only present among human and primate herpesviruses (aa576–584). Low conservation scores were found within the tail-end of the carboxy-terminus of HSV-1 (aa 1048–1123), which are confined to members of the genus simplexvirus.

**Figure 2:**
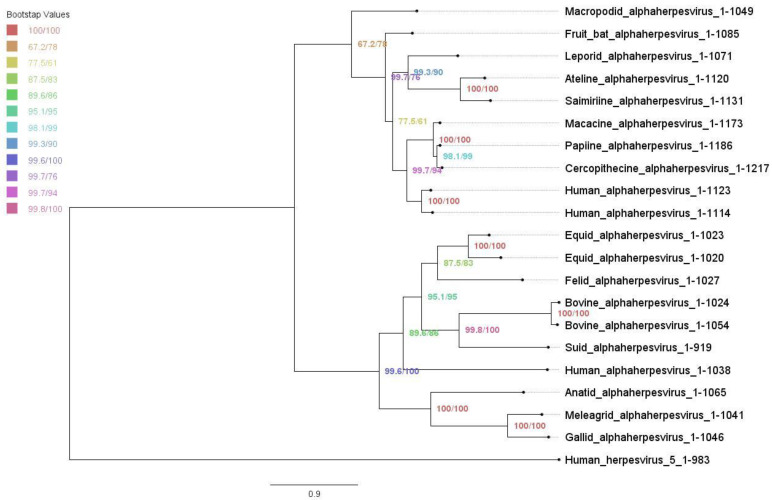
Phylogenetic tree of alphaherpesvirus UL37 orthologs under maximum likelihood. Maximum likelihood tree with ultrafast bootstrapping using IQ-TREE Web Server. Bootstrap values are shown, ranging from 67.2 to 100.

The maximum likelihood tree of UL37 alphaherpesvirus orthologs was constructed using the IQ-TREE Web Server (Figure 2). The overall topology was similar to previously inferred trees based on protein family phylogenetic profiles [52,53]. Bootstrap values showed moderate to good support with a range of 67.2 to 100. Alphaherpesviruses were grouped into two major clades. Simplexviruses were clustered with HSV-1, while members of genus varicellovirus and mardivirus were further grouped into two subclades separating mammalian and avian herpesviruses. The inferred tree was rooted using human herpesvirus-5, which served as an outgroup for reference.

### 3.2. Structural Prediction and Flexibility Analysis of UL37

The complete structures of several tegument proteins have been difficult to resolve using crystallography. This may be due to the structural heterogeneity and proteolytic sensitivity of multi-domain proteins with nonstructured, unfolded regions, making them difficult to express, purify and crystallize [54,55]. Machine-learning strategies with highly accurate outcomes are becoming applicable and impactful for difficult-to-resolve proteins with little experimental data available.

To predict the secondary structure of HSV-1 UL37, we used PSIPRED 4.0, which implements a deep neural network architecture with a general prediction accuracy of 84.2% [35,56]. Results show that HSV-1 UL37 has high alpha-helical content with random coils distributed along the entire amino acid sequence (Supplementary Figure S2). The amino-terminus, central and carboxy-terminus contain long (LDR) and short (SDR) disordered regions.

**Figure 3:**
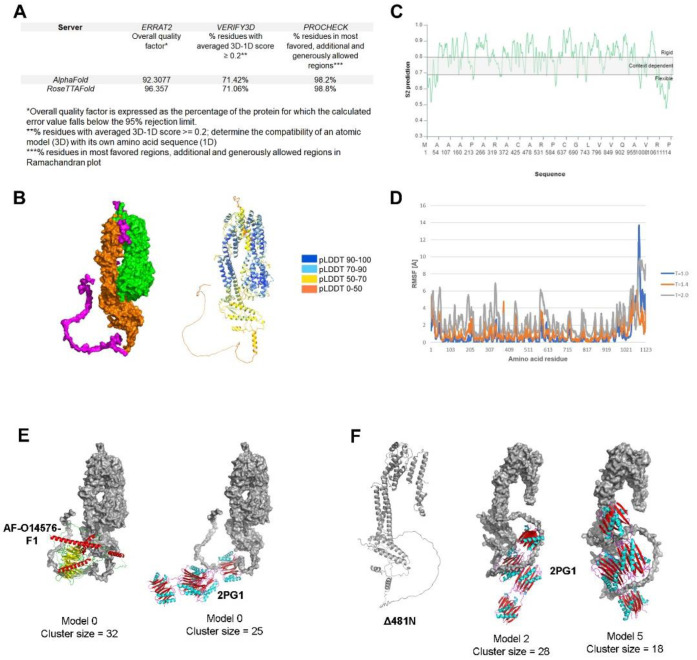
Predicted structure, flexibility and interaction analysis of HSV-1 UL37. (**A**) Evaluation of predicted models generated by RoseTTAFold and AlphaFold. ERRAT2, Verify3D and PROCHECK were used to evaluate the predicted models using the SAVES v6.0 structure validation server (https://saves.mbi.ucla.edu/) (accessed on 17 May 2022) (**B**) AlphaFold predicted model of HSV-1 UL37. Surface model (left) and ribbon model (right) with AlphaFold confidence scores (**C**) Flexibility analysis of HSV-1 UL37. DYNAMINE was used to predict backbone flexibility from sequence (**D**) RMSF analysis of HSV-1 UL37. CABS-Flex 2.0 was used to perform a molecular dynamics simulation for RMSF analysis. (**E**) Protein docking of HSV-1 UL37 with dynein. Docking with human cytoplasmic dynein 1 intermediate chain 1 (AlphaFoldDB Identifier: AF-O14576-F1) (left). Docking with dynein light chain-intermediate chain (LC-IC) complex from Drosophila sp. (PDB ID 2PG1) (right); Dynein intermediate chain models (ribbon models) are bound at the C-terminus of UL37 (surface model). (**F**) Predicted structure of Δ481N and interaction with dynein. AlphaFold2 was used to predict the structure of Δ481N (left). Two models showing interaction of Δ481N with dynein (LC-IC) complex (2PG1) at the C-terminus (right).

To generate the predicted tertiary structure of HSV-1 UL37, we utilized two comparable machine-learning methods, AlphaFold2 and RoseTTAFold. RoseTTAFold generated five models which had similar confidence scores (0.67). We used RoseTTAFold model 1 to compare with the best model produced by AlphaFold2. To determine the overall accuracy and error rate of both models, we subjected the representative models to external quality assessment using ERRAT2, Verify3D and PROCHECK. ERRAT2 calculates an overall quality factor representing the protein percentage with an error value of less than 95%. Error-values are based on the statistical distribution of pairwise atomic interactions, wherein a high distribution of nonbonded atoms represents incorrect structure [36,57]. AlphaFold2 scored an overall quality factor of 92.3077, while RoseTTAFold scored 96.357. Verify3D measures the compatibility of the 3D structure with its amino acid sequence (1D). The percentage of residues with a 3D-1D score above 0.2 should be greater than 80% to be considered a passing score [58]. Both methods failed to reach a score of at least 80%. The percentage of residues with a 3D-1D score of ≥0.2 were 71.42% and 71.06%, for AlphaFold2 and RoseTTAFold, respectively. Ramachandran analysis was performed using PROCHECK, wherein the program classifies allowed and disallowed confirmations based on dihedral angle positions [37,59]. Both methods scored significantly high at 98.2% for AlphaFold2 and 98.8% for RoseTTAFold (Figure 3A).

We examined both models visually and compared them to the secondary structure prediction to determine the final model for further analysis in this study. We found that the overall conformation of both UL37 models from AlphaFold2 and RoseTTAFold was vastly similar, except for the tail end of the carboxy terminus, wherein the RoseTTAFold model attempts to fold the unstructured region (aa 1048–1123) based on secondary structure prediction. Thus, we selected the AlphaFold2 model as the final model for further analysis in this study (Figure 3B).

We mapped out the amino-(UL37N, green) and carboxy-terminus (UL37C, orange) using PyMOL. The overall conformation of UL37 is a clip-like or folded arm-like structure with the two linked, folded domains separated like beads on a string. The SDRs and LDR identified using the predicted secondary structure are depicted in magenta. The centrally located SDR (aa576–584) delineated the amino-and carboxy-terminal regions, so we classified it as a short linker sequence. Interestingly, the centrally located SDR was the same centrally located motif found only in human and primate herpesviruses in our MSA. The predicted structure of UL37N was aligned with the known x-ray crystal structure of HSV-1 (PDB ID 5vyl) (not shown). The predicted structure of UL37N and 5VYL were highly similar to a bean-shaped structure with an RMSD = 0.833. In congruence with the secondary structure prediction, AlphaFold resolved the α-helical core of UL37C, which had not been previously visualized. The UL37 C-terminal half contains two major fibrous alpha-helices of 46–47 amino acids each (aa 735–781; 809–856). UL37C is a mostly folded, elongated domain with a locally disordered tail end (C-tail). The locally disordered C-tail is 75 amino acids in length (aa 1048–1123), which we classified as LDR.

To understand the extent of flexibility of the LDR and SDRs within the UL37 sequence, we examined the amino acid sequence and tertiary structure for predicted backbone flexibility and RMSF analysis (Figure 3C,D). Using the UL37 amino acid sequence as input, we used DynaMine to assess the level of flexibility of the disordered regions. DynaMine calculates the S^2^ prediction value of each amino acid, which can range from 1 (highly rigid) to 0 (highly flexible) [41]. In congruence with the predicted results of DISOPRED3, results show that the disordered regions are variably flexible, with the C-tail (LDR) being more flexible and dynamic (S2 range = 0.4775 to 0.749) as compared to the centrally located SDR (short linker) (S2 range = 0.7512 to 0.7827) and N-terminus (S2 range= 0.5168 to 0.6686; 0.6104–0.6872). The N-terminal and centrally located SDRs appeared to be less flexible than the C-tail LDR.

A previous study has shown that PrV UL37N and UL37C have different melting profiles (61 C˚ and 49 C˚, respectively), which means that each terminal half has distinct thermal stability [60]. Thus, we further investigated how temperature changes affect the entire molecule’s flexibility by subjecting the AlphaFold2 model to a coarse-grained molecular dynamic simulation. Root mean square fluctuation (RMSF) analysis was performed to determine changes in flexibility with increasing temperature. A threshold value of ΔRMSF >0.5 Å based on the default temperature setting (T = 1.4) was used to determine whether the structural movement was significant [43,44]. The CABS-flex 2.0 server generates a plot based on the recorded fluctuations of each amino acid residue during the simulation [43]. We tested the following environments and plotted the results: T = 1.0 (near crystal temperature), T = 1.40 (default temperature) and T = 2.0 (temperature allowing unrestrained protein chains to unfold fully) (Figure 3D). At T = 1.0 (blue), RMSF range values were wide, from 0.024 to 13.712. This range shows that the entire protein appears relatively stable at near crystal temperature, except for the highly flexible C-tail with RMSF values ranging from 2.076 to 13. 712. At T = 1.40 (orange), a narrow range of RMSF values was observed from 0.069 to 6.101. Interestingly, the C-tail at this temperature was less flexible, with RMSF range values from 0.963 to 6.101. At T = 2.0, there was a significant increase in RMSF values within the entire UL37 amino acid sequence, with RMSF values ranging from 0.602 to 9.823. These results show that increasing temperature alters the flexibility of specific UL37 amino acid residues.

#### 3.2.1. UL37 Domains Predicted to Interact with Dynein

To determine whether a binding partner of UL37 interacts with any of the potential binding sites of UL37, we performed a protein docking simulation using ClusPro 2.0. We selected the dynein light chain-intermediate chain (LC-IC) complex (PDB ID 2PG1) and the predicted structure of the human cytoplasmic dynein 1 intermediate chain 1 (AlphaFoldDB Identifier: AF-O14576-F1) to test interaction with the HSV-1 UL37 model. Previously, our laboratory has shown that UL37 interacts with the dynein IC to facilitate retrograde intracellular transport of the viral capsid to the nucleus [19]. Protein docking models show potential interaction of dynein (cartoon model) at different contact points of the C-terminus of UL37 (surface model) (Figure 3E).

#### 3.2.2. The Effect of the Δ481N Truncation on the Predicted Structure of the UL37 C-Terminus

The predicted structure of Δ481N revealed that the remaining portion of the protein was not appreciably affected. A small region of the N-terminus remained in a folded arm-like conformation, and the C-terminal domain maintained its elongated structure. Protein docking of the Δ481N UL37 to dynein (2PG1) demonstrated preserved interaction between the two proteins at the C-tail end (Figure 3F).

### 3.3. Predicted Functional Attributes of the UL37 Protein

#### 3.3.1. Hydrophobicity, Electrostatic Potential, and Conservation Analysis of UL37

**Figure 4:**
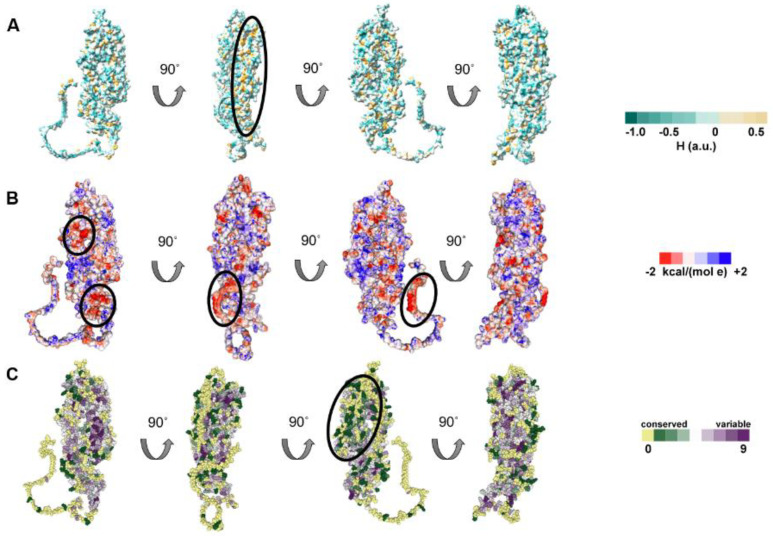
Surface analysis of HSV-1 UL37. (**A**) Hydrophobicity of HSV–1 UL37. HSV–1 UL37 tegument protein is predominantly hydrophilic with hydrophobic pockets. (**B**) Electrostatic potential of HSV–1 UL37. Negatively charged prominences are encircled. (**C**) Conservation analysis of HSV–1 UL37. HSV–1 UL37 consists of a conserved core and variable surface (encircled).

UL37 surface hydrophobicity was examined using ChimeraX. The visualization shows that most surface amino acids were hydrophilic with pockets of hydrophobic regions (Figure 4A). Hydrophobic pockets were prominent on the posterior part of UL37C (encircled). The surface electrostatic potential of HSV-1 UL37 shows negatively charged prominences, commonly distributed on the C-terminal half. (Figure 4B). The ConSurf web server allows visualization of the level of conservation of each amino acid by estimating the evolutionary rates of a query sequence relative to its homologs [45]. After using the AlphaFold model as input, it was possible to map variable and conserved regions in HSV-1 UL37 (Figure 4C). The inner helical core of both UL37N and UL37C is composed of highly conserved amino acid residues. On one side of the protein, surface residues on UL37N were highly variable (encircled), while conservation was present on the opposite side, extending into the core of the structure (Figure 4C). Taken together, these highly conserved and highly variable, negatively charged, and hydrophobic regions indicate possible interaction sites on the surface of HSV-1 UL37.

#### 3.3.2. Conservation of the UL37 Structure in Other Alphaherpesviruses

The HSV-1 virus is the prototypic alphaherpesvirus. To extend our modeling results to other alphaherpesviruses, we analyzed the predicted structure of varicellovirus orthologs, bovine herpesvirus 1 (BoHV-1) and pseudorabies (PrV) UL37 (Supplementary Figure S3). The overall folded arm-like conformation was strikingly similar for HSV-1, PrV and BoHV-1. The two flexible, disordered regions at the PrV and BoHV-1 C-tail were short and spaced with alpha helices, unlike the HSV-1 UL37 C-tail, which was significantly extended and uninterrupted by any helices. Thus, although these UL37 proteins exhibited a high degree of structural conservation, specific differences may be attributed to UL37 host species-specific functions.

#### 3.3.3. Tegument Protein UL37 Is Predicted to Act as a Peripheral Membrane Protein with Potential Interaction Domains in the C-Tail

**Figure 5:**
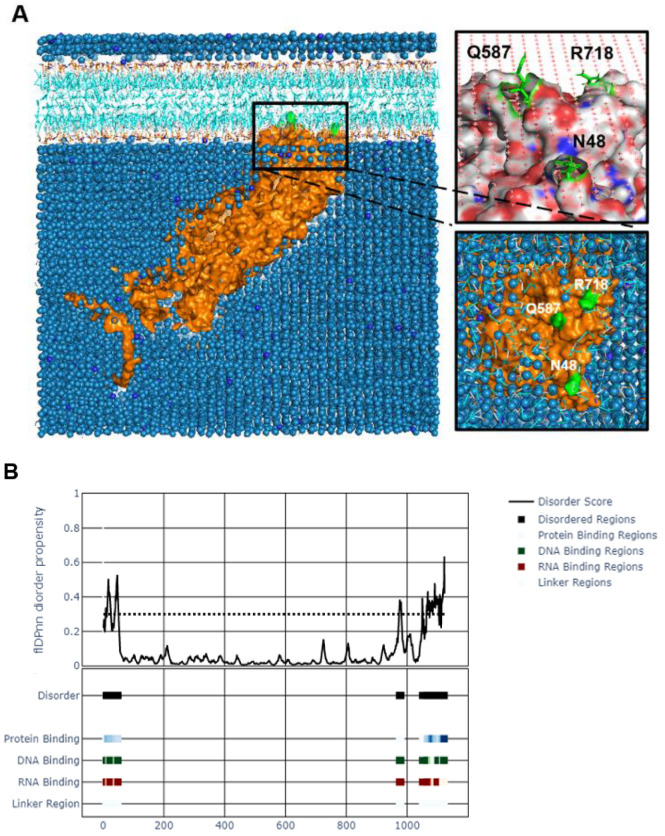
Interaction analysis of tegument protein UL37. (**A**). Molecular dynamics simulation of HSV-1 UL37 bilipid membrane interaction using MemProtMD. HSV-1 UL37 is predicted to be a peripheral protein interacting with the inner layer of the bilipid membrane. (**B**). Potential DNA, RNA and protein binding regions within disordered N-and C-termini. Binding sites were identified within the disordered regions using flDPnn.

As a tegument protein with known interactions with membrane proteins gK and UL20, we wanted to know how UL37 interacts with bilipid membranes. We performed a coarse-grained molecular dynamics simulation using MemProtMD to attempt the insertion of UL37 into a lipid bilayer. We found that UL37 interacts with the inner layer of the lipid membrane by three contact points found in both UL37N and UL37C (Figure 5A). Two residues, asparagine (N48) and glutamine (Q587), are uncharged and in contact with the inner lipid membrane. Interestingly, these are found shortly after the SDRs on the N-terminus and central locations. Similarly, an arginine residue (R718) interacted with the lipid layer. This positively charged residue is found on UL37C.

The potential function of the unstructured regions was of interest because of their intrinsic disorder, flexibility, high variability, and accessibility on the surface of the entire protein. IDRs within a protein are often attributed to multiple functions. We used a recently published computational tool, flDPnn, which predicts the four most common disorder functions in an IDR by the random forest (RF) method [42]. flDPnn is the only computational tool that predicts putative functions of disordered regions. It calculates protein, DNA, and RNA-binding propensities ranging from 0 to 1, with higher values indicating more accurate predictions. flDPnn identified specific amino acids within the unstructured N-and C-termini as protein-, DNA-and RNA-binding regions with predictive values at 1.0. Specific amino acids identified as protein-, DNA-, and RNA-binding sites are listed in Supplementary Table S3.

### 3.4. Viral Replication Kinetics and Plaque Morphology of the Δ481N Virus

**Figure 6:**
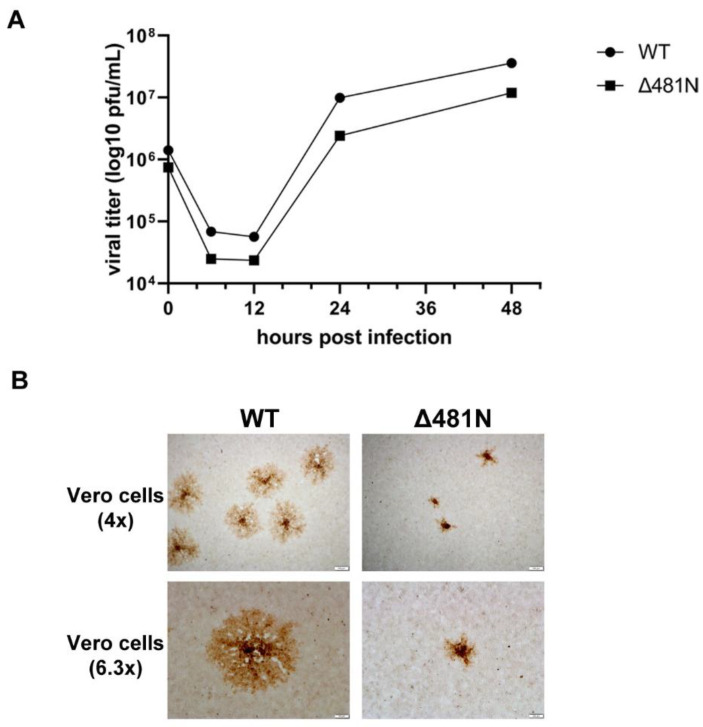
Viral replication kinetics and plaque morphology of wildtype and Δ481N mutant virus. (**A**) Viral replication curve. Viral replication curve of Δ481N mutant virus shows similar growth kinetics to wildtype. (**B**) Plaque morphology. Significant smaller plaque formation in Δ481N mutant virus as compared to wildtype.

The 480 amino acid deletion was the only deletion that allowed the virus to replicate in cell culture among various amino-terminal and carboxyl-terminal truncations generated by the Desai laboratory [23]. To examine the replication properties of Δ481N mutant virus, we investigated its viral replication kinetics and plaque morphology. Vero cells were infected with either WT or Δ481N mutant virus at MOI = 1, and collected at 0, 6, 12, 24 and 48 hpi. The Δ481N virus replicated efficiently compared to WT virus in Vero cells (Figure 6A). However, the Δ481N virus produced significantly smaller viral plaques indicating a defect in virion egress and spread (Figure 6B). These results are consistent with our previous work indicating that the HSV-1 UL37 protein interacts with the gK/UL20 protein complex and functions in efficient virus envelopment in the TGN and subsequent egress out of infected cells [61].

### 3.5. The Effect of the Δ481N UL37 Deletion in Virus Infection and Retrograde Transport

**Figure 7:**
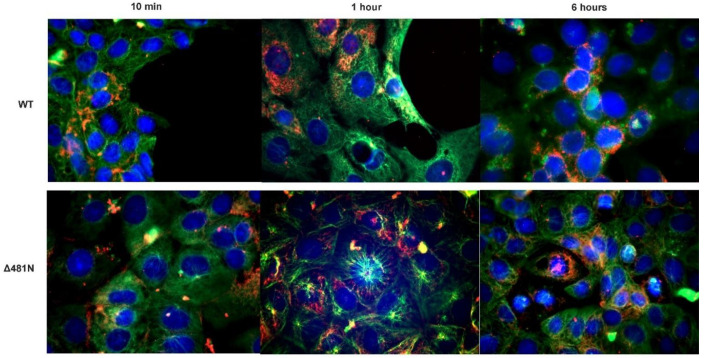
Intracellular transport assay of WT and Δ481N mutant virus. Vero cells were infected with WT and Δ481N mutant virus and examined for transport of viral capsids (red) on the tubulin network (green) at 10 min, 1 h and 6 h after adsorption. Rabbit α-tubulin (Abcam, Inc., Cambridge, MA, USA) (1:100) and mouse anti-HSV-1 ICP5 (Santa Cruz Biotechnology Dallas, TX, USA) (1:100) were used to visualize viral capsid transport. Secondary antibodies used were anti-rabbit Alexa Fluor^TM^ 488 anti-mouse Alexa Fluor^TM^ 647 (1:1000), respectively. Nuclei (blue) were stained using DAPI (49,6-diamidino-2-phenylindole).

To examine the effect of the Δ481N deletion in virion infectivity and retrograde transport, nearly confluent Vero cell monolayers were infected with an MOI of 50 with wildtype or the Δ481N virus. At 10 min, 1 h and 6 h post-infection, cells were fixed and stained with anti_VP5 antibody to detect the virion capsids, anti-tubulin antibody to visualize the tubulin network and DAPI to visualize the nuclei of infected cells. Both viruses constitutively express GFP fused in-frame at the carboxyl terminus of the UL37 protein. Immunofluorescence images of the infected cells revealed that both viruses were able to attach and be transported in a retrograde manner to the nuclei of infected cells with similar kinetics (Figure 7). Similar experiments were performed using SH-SY5Y cells in microfluidic devices to investigate whether the Δ481N virus could be infected and transported in a retrograde manner to the nuclei of infected neurons. Fluorescence imaging revealed that both viruses were efficiently transported in a retrograde manner in neurons (Supplementary Figure S4).

## 4. Discussion

### 4.1. Functional Domains of the HSV-1 UL37 Protein

The HSV-1 UL37 tegument protein contains important functional domains within the amino and carboxyl termini. However, the functional properties of the proximal, central, and distal ends of the UL37 amino acid sequence remain undetermined. The complete structure of HSV-1 UL37 has been difficult to resolve by x-ray crystallography. In this paper, we analyzed the entire alphaherpesvirus UL37 tegument protein secondary and tertiary structure, flexibility, intrinsic disorder, surface and evolutionary characteristics, membrane interaction and potential binding sites using multiple computational approaches. Herein, we show that HSV-1 UL37 is predicted to be a globular, multi-domain protein with the N-(aa1–575) and C-termini (585–1123) as two distinct domains connected by a short linker sequence (aa576–584), with a locally disordered C-tail (aa1048–1123). The predicted tertiary structure reveals that the alpha-helical core of UL37N and UL37C assumes a folded arm-like conformation. Based on this predicted model, we examine the effect of deleting the first 480 amino acids of the HSV-1 UL37 protein on its predicted structure and its function in virus infection and retrograde transport. Results showed that the predicted structure of the remaining portion of the UL37 protein was largely unaffected. In agreement with this prediction, we show that the mutated UL37 protein allowed efficient virus infection and retrograde transport in cells and neurons, indicating that the C-terminal portion of the HSV-1 UL37 protein contains necessary and sufficient domains for engaging the dynein motor complex for retrograde transport of virion capsids to the nuclei of infected cells.

### 4.2. Structure of the UL37 Amino Terminus

The crystal structure of the N-terminal half of HSV-1 UL37 is a single chain with a modeled residue count of 502 amino acids and a deposited residue count of 578 amino acids due to unresolved sequences in the proximal end of the N-terminus. It was previously described as a rigid, bean-shaped structure with an alpha-helical core using x-ray crystallography; however, the crystal structure of the C-terminal half could not be determined despite exhaustive efforts [62,63]. As a result, the shape and flexibility of PrV UL37C were determined using small angle x-ray scattering (SAXS) modeling [60]. Using SAXS data, UL37C was described as having an elongated, folded core with an unstructured C-tail. The predicted structure of UL37C shown in this paper corresponds with the elongated shape depicted by the SAXS model; however, this is the first time the ordered region of the C-terminal half and the folded arm-like position of the N-and C-terminal halves have been visually captured. We propose that the folded arm-like conformation allows the protein to anchor itself to a lipid membrane while exposing surfaces to binding partners on either side of the N-and C-terminal half and at the tail end of the C-terminus. Our findings on the predicted structure of UL37 support the ability of UL37 to bind different proteins and nucleic acids, potentially, in a regulated manner, at different locations or stages of the replication cycle.

### 4.3. Structure of the UL37 Carboxy Terminus 

The structure of the C-terminal half of the UL37 protein is of significant interest due to the multiple interactions found in this primarily flexible region. In a previous study, dimensionless Kratky plots of PrV UL37C had varied profiles, indicating that UL37C is conformationally flexible, containing equal amounts of ordered and disordered regions [60]. The AlphaFold2 predicted model of UL37 provides a granular visualization of the ordered region of the C-terminal half, which significantly complements the SAXS model from the aforementioned study. The folded alpha-helical core of the C-terminal half begins at amino acid 592, which coincides with the secondary structure prediction. Two fibrous alpha-helices of 46–47 amino acids within UL37C (aa 735–781; 809–856) make up the elongated core of UL37. Interestingly, it is similar to the partially resolved filamentous structure of HSV-1 UL36 (central 970 residues) [64], which interacts with UL37 within the viral tegument and in infected cells [65,66]. Moreover, this elongated structure is important for immunoregulation as it contains the active sites for RIG-I and cGAS deamidation [20,22]. Using the ensemble optimization method (EOM), it has been suggested that UL37C can adopt several conformations with short and long forms, alluding to a controlled binding capability [60]. We show that with increasing temperature, RMSF values increase significantly, indicating unfolding, especially at the C-tail. Several active and passive environmental factors can influence the process of local unfolding within a protein [67]. Disordered regions may unfold due to passive factors such as exposure to other substrates; alteration of the environment-such as transport within cellular compartments; and changes in pH, temperature, or redox potential. Active factors include membrane interaction, protein and nucleic acid binding, and post-translational modifications [67]. In some cases, the functionality of a protein is activated during a conformational change from being folded to unfolded [68]. The significant shift in RMSF in the C-terminal half indicates enzyme thermal stability [69]. HSV-1 UL37 is a viral deamidase that can regulate the host immune response [35,59]. Flexible conformations found within a conserved region of a virus have been correlated with immune evasion, as in hepatitis C virus and HIV [70,71]. Our findings on the predicted structure of UL37 support the previous findings on the rigid and flexible nature of UL37C.

### 4.4. Functional Domains within the UL37 Carboxy Terminus 

The HSV-1 unstructured C-tail has been predicted to bind DNA, RNA, and proteins. We have previously shown that UL37 binds the dynein IC motor protein to facilitate retrograde and anterograde transport [19]. We predict that the dynein IC binds at multiple points within the unstructured UL37 C-tail (aa1048–1123). Based on the evidence of a nuclear export signal at the N-terminus, it has been previously suggested that HSV-1 UL37 acts as an RNA transport protein; however, RNA binding motifs could not be found computationally at that time [14]. With current knowledge of the binding capability of disordered regions, we predicted the potential RNA-binding sites within the disordered C-tail computationally. Purified herpesvirus virions have been previously found to contain viral and cellular transcripts [72]. Other tegument proteins, such as the membrane-associated UL11 and capsid-binding UL21, have been shown to possess RNA-binding capability [73,74]. Although it has not been shown that these tegument proteins are involved in packaging viral or cellular transcripts, it is possible that the UL37 unstructured C-tail, together with other RNA-binding tegument proteins, play an important role in packaging viral or cellular RNA during envelopment. Previously, it has been shown that the DNA-binding capability of UL37 is dependent on the presence of ICP8 protein [75,76]. It is possible that the unstructured C-tail also facilitates this supportive function of UL37. Thus, the UL37 C tail may have both DNA-and RNA-binding functions.

### 4.5. Potential Functions of the “Hinge” Domains Separating Amino-Terminal and Carboxyl-Terminal Domains

A hinge or linker often separates polypeptide chains that are folded onto themselves. The disordered 9 amino acid sequence separating the N-and C-terminal halves are composed of A-T-P-L-S-A-L-L-P. Proline (Pro), threonine (Thr), alanine (A), and serine (S) are considered to be preferred amino acids in natural protein linkers [77,78]. Protein linkers may either be α-helices or coils/bends and act as connectors of multi-domain proteins. In the predicted structure of HSV-1 UL37, it appears to be within a short, disordered coil. Protein linkers may serve biological functions such as increasing flexibility of separated domains, facilitating interaction or preventing interference between multiple domains and can be classified as flexible or rigid [77,79]. Based on the low predicted flexibility of this region using DynaMine, we propose that the identified linker acts as a rigid spacer to prevent the interaction of the N-and C-terminal halves. In PrV UL37, UL37N and UL37C are independent domains with no interaction [60]. Identification of the short linker sequence defines the UL37N and UL37C domains and provides insights into the individual functionality of the two protein domains. Lastly, future studies may address the difficulty of expressing UL37C as a separate epitope by beginning at the point of helix formation, which may prevent unfolding and enhance the probability of preserving the helical core structure of UL37C.

### 4.6. Domains of the UL37 Protein That Function in Virion Intracellular Transport 

Functional studies with the Δ481N virus indicated that this UL37 deletion did not affect the intracellular transport of virion capsids to the nuclei of infected cells. In this instance, this result is congruent with the conservation of the predicted C-terminal structure of the Δ481N UL37 protein. Previous studies have indicated that multiple mutations in the UL37 protein inhibited intracellular transport rendering the virus avirulent in mice [15]. Based on the location of these disparate mutations distributed throughout the UL37 protein, we predict that only two mutations that map in the C-terminus may function in intracellular transport.

### 4.7. Functions of UL37 in Virion Morphogenesis and Egress 

Many earlier studies on UL37 function were focused on its role in virion morphogenesis, specifically, intracellular trafficking pathways involving viral transport to and from the nucleus towards the TGN via microtubules. Significant differences exist in the requirement of tegument protein UL37 in HSV-1 and PrV. Both mutant viruses of HSV-1 and PrV without UL37 expression are severely delayed in the transport of capsids towards the nucleus and, subsequently, transport towards the sites of secondary envelopment, i.e., TGN [80,81]. In HSV-1, UL37 is necessary for productive viral replication, unlike in PrV, where PrV-ΔUL37 mutant viruses are productive, albeit at lower titers than the wildtype [80]. As a membranous compartment, the TGN lipid composition is mostly sphingolipids and cholesterol, which are similar attributes of the plasma membrane (PM) [82,83]. Our CGMD simulation for lipid interaction shows that UL37 interacts with lipid bilayers as a peripheral membrane protein (PMP), which interacts via the polar head groups of phospholipids. We classify UL37 as a PMP as no structures were found within UL37 that totally or partially integrated with the lipid membrane except for three contact points, N48, Q587 and R718. We attribute the predicted UL37-lipid membrane interaction with the functional distribution of UL37 within the TGN. As the site of secondary envelopment, UL37 is essential in directing viral capsids to the TGN [81]. Furthermore, UL37 migrates to the TGN without the viral capsid, together with the major tegument protein UL36 [84]. We suspect that UL37 potentially stabilizes the UL36/UL37 complex within the TGN via this lipid interaction. Peripheral membrane proteins (PMP) may be divided into two classifications: PMPs that associate with the membrane thru electrostatic or hydrophobic interactions and PMPs which attach themselves to the membrane using a hydrophobic segment without integration [85]. The UL37-lipid membrane interaction may be electrostatic due to the nature of the amino acids in contact with the inner layer, especially R718, which is a positively charged residue found within the C-terminal half. The surface potential of the inner layer of lipid membranes leads to the attraction of positively charged proteins, motifs, and ions [86]. PMP association with lipid membranes is reversible via changes in pH or concentration [85]. As a gamma 1 class gene, UL37 accumulates during late infection, potentially increasing its concentration and probable association with the lipid membrane. We hypothesize that as a PMP, UL37 potentially performs multiple functions, including capsid traffic control, secondary envelopment, and immunomodulation, by associating and disassociating with the TGN. Therefore, other supportive functions of HSV-1 UL37 may depend on its ability to interact with lipid membranes, which remains to be further explored. Site-directed mutagenesis in conjunction with *in silico* docking experiments may be able to resolve functional domains of the UL37 protein further.

### 4.8. Prediction and Comparisons of HSV-1 with Other Alphaherpesvirus UL37 Proteins 

Based on our MSA and evolutionary conservation analysis, the unstructured C-tail (aa1048–1123) of HSV-1 UL37 appears to be the most extended and highly variable among *simplexviruses* and *varicelloviruses* at 75 amino acids, unlike the inner helical core, which was highly conserved. These results may signify that the UL37 core is maintained evolutionarily for the structural stability of the protein, while the exposed regions are diversified and adaptive to the host. We analyzed the predicted structure of a *varicellovirus* ortholog, BoHV-1 and PrV UL37. The two flexible, disordered regions at the BoHV-1 C-tail were short and spaced with alpha helices (aa923–932; 1020–1026), unlike the HSV-1 UL37 C-tail, which was significantly extended and uninterrupted by any helices. Interestingly, truncation of the C-tail of HSV-1 (133 amino acids) is lethal to the virus, unlike a PrV UL37-null virus, which was previously described as capable of productive replication [23,79]. These differences suggest that the HSV-1 UL37 C-terminus has evolved to provide additional important functions in virus infection.

## 5. Conclusions

The predicted structure of HSV-1 UL37 tegument protein demonstrates the importance of the flexible C-terminal half, especially the unstructured C-tail. These results indicate that the amino terminus of UL37 proteins may be more critical in virus assembly and egress out of infected cells, while the most important functions for viral infectivity and intracellular transport and specified by the C-tail of the UL37 protein. Further confirmatory analysis on the functions of the C-terminal half and the unstructured C-tail is necessary.

## Data Availability

The data presented in this study are available within the article or supplementary material. The PDB files of predicted structures presented in this study are available on request from the corresponding author.

## Supplementary Materials

The following supporting information can be downloaded at: https://www.mdpi.com/article/10.3390/v14102189/s1, Table S1: Web-based tools and software used in this study; Table S2: Alphaherpesvirus UL37 orthologs arranged by subfamily used in the phylogenetic analysis; Figure S1. Multiple sequence alignment (MSA) using ClustalO in Jalview; Table S3: Specific amino acid residues in HSV-1 UL37 with protein-, RNA-and DNA-binding potential. Figure S2. Predicted secondary structure and disordered regions in HSV-1 UL37 using PSIPRED 4.0 and DISOPRED3; Figure S3. Predicted structures of HSV-1, PrV and BoHV-1 UL37; Figure S4. Retrograde transport of WT and Δ481N mutant virus.
