# Supplementary material for: Predicted Structure and Functions of the Prototypic Alphaherpesvirus Herpes Simplex Virus Type-1 UL37 Tegument Protein
Source: Viruses. 2022 Oct 4;14(10):2189. doi: 10.3390/v14102189 (PMC9608200; doi:10.3390/v14102189)
Supplement: Supplementary file 1 [file viruses-14-02189-s001.zip › Supplemental Table S1.pdf]

Supplemental Table 1. Web-based tools and software used in this study. The following toolbox for structural analysis was created for this study.

Server Name	Active link
Jalview	https://www.jalview.org/
IQ-TREE Web Server	http://iqtree.cibiv.univie.ac.at/
Protein BLAST	https://blast.ncbi.nlm.nih.gov/Blast.cgi
PSIPRED 4.0	http://bioinf.cs.ucl.ac.uk/psipred/
DISOPRED3	http://bioinf.cs.ucl.ac.uk/psipred/
ERRAT2	https://saves.mbi.ucla.edu/
Verify3D	https://saves.mbi.ucla.edu/
PROCHECK	https://saves.mbi.ucla.edu/
AlphaFold2	https://colab.research.google.com/github/sokrypton/ColabFold/blob/main/AlphaFold2.ipynb
RoseTTAFold	https://robetta.bakerlab.org/
DynaMine	https://bio2byte.be/dynamine/#
flDPnn	http://biomine.cs.vcu.edu/servers/flDPnn/
MemprotMD	https://colab.research.google.com/github/pstansfeld/MemProtMD/blob/main/MemProtMD_Insane.ipynb
CABS-flex 2.0	http://biocomp.chem.uw.edu.pl/CABSflex2
UCSF ChimeraX	https://www.cgl.ucsf.edu/chimerax/
ConSurf	https://consurf.tau.ac.il/
PEBQ Solver	https://charmm-gui.org/
ClusPro 2.0	https://cluspro.bu.edu/
