# Supplementary material for: Predicted Structure and Functions of the Prototypic Alphaherpesvirus Herpes Simplex Virus Type-1 UL37 Tegument Protein
Source: Viruses. 2022 Oct 4;14(10):2189. doi: 10.3390/v14102189 (PMC9608200; doi:10.3390/v14102189)
Supplement: Supplementary file 1 [file viruses-14-02189-s001.zip › Supplemental Table S2.pdf]

Supplemental Table 2. Alphaherpesvirus UL37 orthologs arranged by subfamily used in phylogenetic analysis. A standard protein BLAST search (https://blast.ncbi.nlm.nih.gov/Blast.cgi) was performed using AFP86401.1 as the query sequence within Alphaherpesvirinae (taxid 10293) organisms and Reference Proteins (refseq_protein) as database.

Scientific name	Accession Number	% Identity with HSV-1
**Simplexviruses**		
Human alphaherpesvirus 1	YP_009137112.1	100%
Human alphaherpesvirus 2	YP_009137189.1	84.68%
Macacine alphaherpesvirus 1	NP_851897.1	71.85%
Papiine alphaherpesvirus 2	YP_443884.1	71.40%
Cercopithecine alphaherpesvirus 2	YP_164480.1	71.03%
Fruit bat alphaherpesvirus 1	YP_009042099.1	64.24%
Leporid alphaherpesvirus 4	YP_009230167.1	58.60%
Ateline alphaherpesvirus 1	YP_009361900.1	56.60%
Saimiriine alphaherpesvirus 1	YP_003933802.1	51.46%
Macropodid alphaherpesvirus 1	YP_009227270.1	45.62%
**Varicelloviruses**		
Equid alphaherpesvirus-3	YP_009054926.1	31.48%
Equid alphaherpesvirus1	YP_053068.1	28.96%
Felid alphaherpesvirus 1	YP_003331542.2	27.65%
Bovine alphaherpesvirus 1	NP_045321.1	29.50%
Bovine alphaherpesvirus 5	NP_954910.1	30.37%
Suid alphaherpesvirus 1	YP_068340.1	28.53%
Human alphaherpesvirus 3	NP_040144.1	27.86%
**Mardiviruses**		
Anatid alphaherpesvirus 1	YP_003084384.1	28.37%
Meleagrid alphaherpesvirus 1	NP_073331.1	25.20%
Gallid alphaherpesvirus 2	YP_001033966.1	26.71%
