# Supplementary material for: Predicted Structure and Functions of the Prototypic Alphaherpesvirus Herpes Simplex Virus Type-1 UL37 Tegument Protein
Source: Viruses. 2022 Oct 4;14(10):2189. doi: 10.3390/v14102189 (PMC9608200; doi:10.3390/v14102189)
Supplement: Supplementary file 1 [file viruses-14-02189-s001.zip › Supplemental Table S3.pdf]

Supplemental Table 3. Specific amino acid residues in HSV-1 UL37 with protein-, RNA- and DNA-binding potential. Predicted disordered amino acid residues with protein-, DNA-, and RNA- binding scores ranging from 0.300 to 1.000.

Disordered amino acid residue	Protein binding score	DNA binding score	RNA binding score
**E9**	0.365	1.000	0.947
**A10**	0.347	1.000	0.890
**T14**	0.712	1.000	0.804
**T15**	0.712	1.000	0.783
**S16**	0.719	1.000	0.783
**P17**	0.719	1.000	0.723
**A18**	0.719	0.465	0.494
**G19**	0.697	0.465	0.533
**P20**	0.733	0.465	0.533
**P21**	0.726	0.465	0.533
**S22**	0.727	1.000	0.764
**D23**	0.707	1.000	0.773
**G24**	0.708	1.000	0.805
**P25**	0.684	1.000	0.816
**P39**	0.659	1.000	0.762
**P40**	0.660	1.000	0.761
**T41**	0.660	1.000	0.738
**P42**	0.660	1.000	0.791
**T43**	0.660	0.465	0.529
**A44**	0.582	0.465	0.498
**E45**	0.614	0.465	0.503
**T46**	0.671	0.465	0.494
**A47**	0.672	1.000	0.725
**N48**	0.647	1.000	0.771
**G49**	0.647	1.000	0.769
**A50**	0.604	1.000	0.760
**P974**	0.374	1.000	0.826
**P975**	0.375	1.000	0.781
**Q976**	0.375	1.000	0.760
**S977**	0.375	1.000	0.781
**P978**	0.375	1.000	0.767
**A979**	0.375	1.000	0.762
**A980**	0.360	1.000	0.773
**A1050**	0.305	1.000	0.911
**G1051**	0.300	1.000	0.932
**G1064**	0.512	1.000	0.938
**S1066**	0.645	1.000	1.000
**L1067**	0.645	1.000	0.967
**P1068**	0.645	1.000	0.959
**A1069**	0.645	1.000	0.980
**P1070**	0.657	1.000	0.972
**M1071**	0.665	1.000	0.965
**P1072**	0.668	1.000	0.960

**M1073**	0.668	1.000	0.939
**Q1076**	0.683	1.000	0.965
**K1077**	0.683	1.000	0.954
**P1078**	0.683	1.000	0.919
**E1079**	0.731	1.000	0.649
**A1080**	0.765	0.465	0.656
**Y1081**	0.778	0.465	0.643
**G1082**	0.802	0.465	0.664
**H1083**	0.815	0.465	0.664
**G1084**	0.896	0.465	0.640
**P1085**	0.896	0.465	0.557
**R1086**	0.862	0.465	0.619
**Q1087**	0.864	0.465	0.585
**A1088**	0.986	0.300	0.519
**D1089**	0.934	0.300	0.492
**R1090**	0.816	0.300	0.463
**E1091**	0.827	0.300	0.463
**G1092**	0.828	0.300	0.500
**A1093**	0.784	0.300	0.513
**P1094**	0.779	0.300	0.492
**H1095**	0.715	0.300	0.513
**S1096**	0.691	0.300	0.492
**N1097**	0.705	0.300	0.564
**T1098**	0.688	0.300	0.607
**P1099**	0.683	0.300	0.628
**V1100**	0.668	0.835	0.928
**E1101**	0.668	0.835	0.928
**D1102**	0.668	0.835	0.907
**D1103**	0.668	0.835	0.914
**G1064**	0.512	1.000	0.938
**S1066**	0.645	1.000	1.000
**L1067**	0.645	1.000	0.967
**P1068**	0.645	1.000	0.959
**A1069**	0.645	1.000	0.980
**P1070**	0.657	1.000	0.972
**M1071**	0.665	1.000	0.965
**P1072**	0.668	1.000	0.960
**M1073**	0.668	1.000	0.939
**Q1076**	0.683	1.000	0.965
**K1077**	0.683	1.000	0.954
**P1078**	0.683	1.000	0.919
**E1079**	0.731	1.000	0.919
**A1080**	0.765	0.465	0.649
**Y1081**	0.778	0.465	0.656
**G1082**	0.802	0.465	0.643
**H1083**	0.815	0.465	0.664
**G1084**	0.896	0.465	0.664
**P1085**	0.896	0.465	0.640
**R1086**	0.862	0.465	0.557

**Q1087**	0.864	0.465	0.619
**A1088**	0.986	0.300	0.585
**D1089**	0.934	0.300	0.519
**R1090**	0.816	0.300	0.492
**E1091**	0.827	0.300	0.463
**G1092**	0.828	0.300	0.500
**A1093**	0.784	0.300	0.513
**P1094**	0.779	0.300	0.492
**H1095**	0.715	0.300	0.513
**S1096**	0.691	0.300	0.492
**N1097**	0.705	0.300	0.564
**T1098**	0.688	0.300	0.607
**P1099**	0.683	0.300	0.628
**V1100**	0.668	0.835	0.928
**E1101**	0.668	0.835	0.928
**D1102**	0.668	0.835	0.907
**D1103**	0.668	0.835	0.914
**E1108**	0.525	0.525	0.877
**D1109**	0.532	0.532	0.900
**V1111**	0.672	0.672	0.806
**P1113**	0.664	0.664	0.757
**P1114**	0.667	0.667	0.723
**T1115**	0.667	0.667	0.762
**D1116**	0.681	0.681	0.323
**L1117**	0.681	0.681	0.323
**P1118**	0.670	0.670	0.308
**L1119**	0.848	0.848	0.300
**T1120**	0.848	0.848	0.317
**S1121**	0.863	0.863	0.307
**Y1122**	0.995	0.995	0.361
**Q1123**	1.000	1.000	0.409
