# Supplementary figures and images for: Predicted Structure and Functions of the Prototypic Alphaherpesvirus Herpes Simplex Virus Type-1 UL37 Tegument Protein
Source: Viruses. 2022 Oct 4;14(10):2189. doi: 10.3390/v14102189 (PMC9608200; doi:10.3390/v14102189)

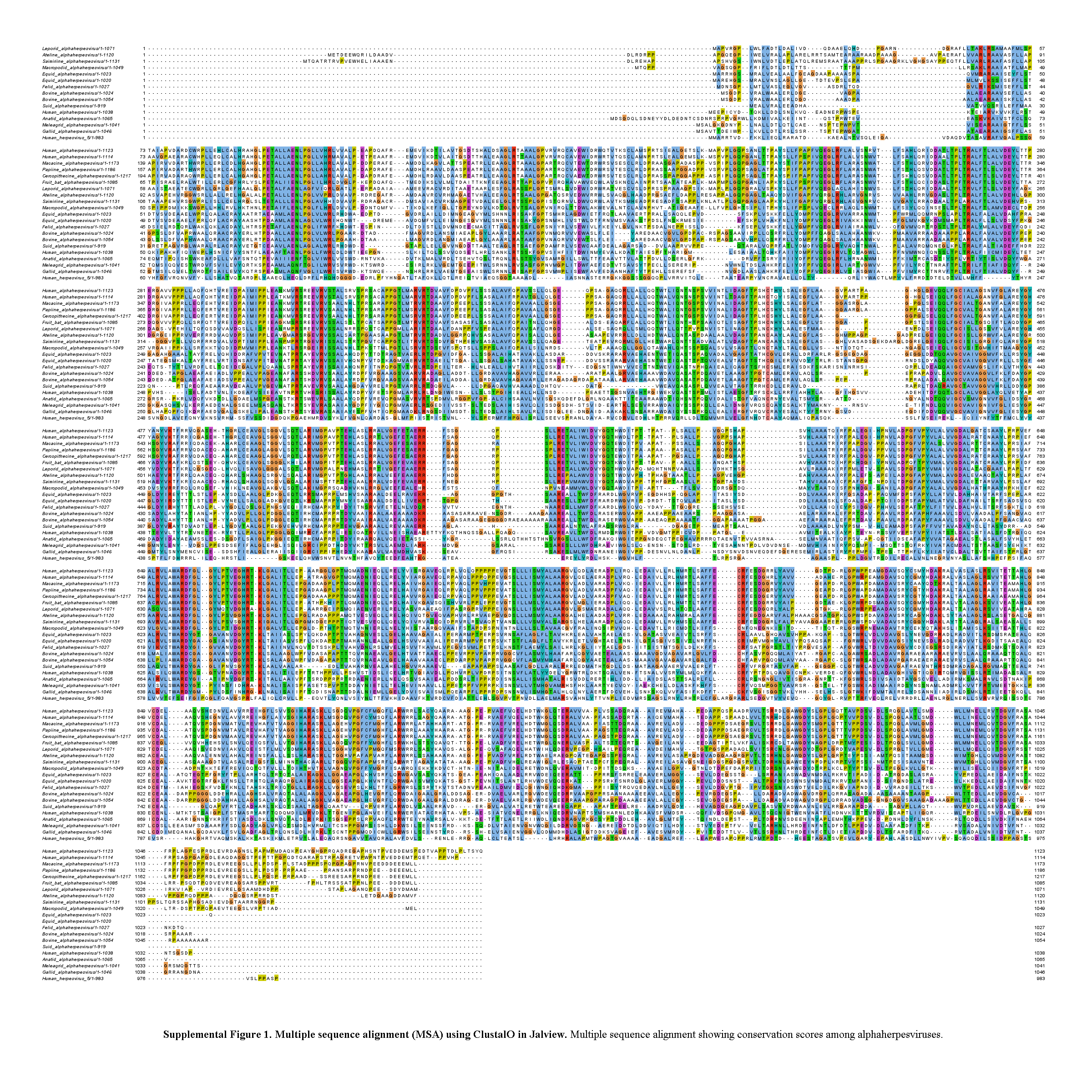

Supplement: Supplementary file 1 [file viruses-14-02189-s001.zip › Supplemental Figure S1.jpg]

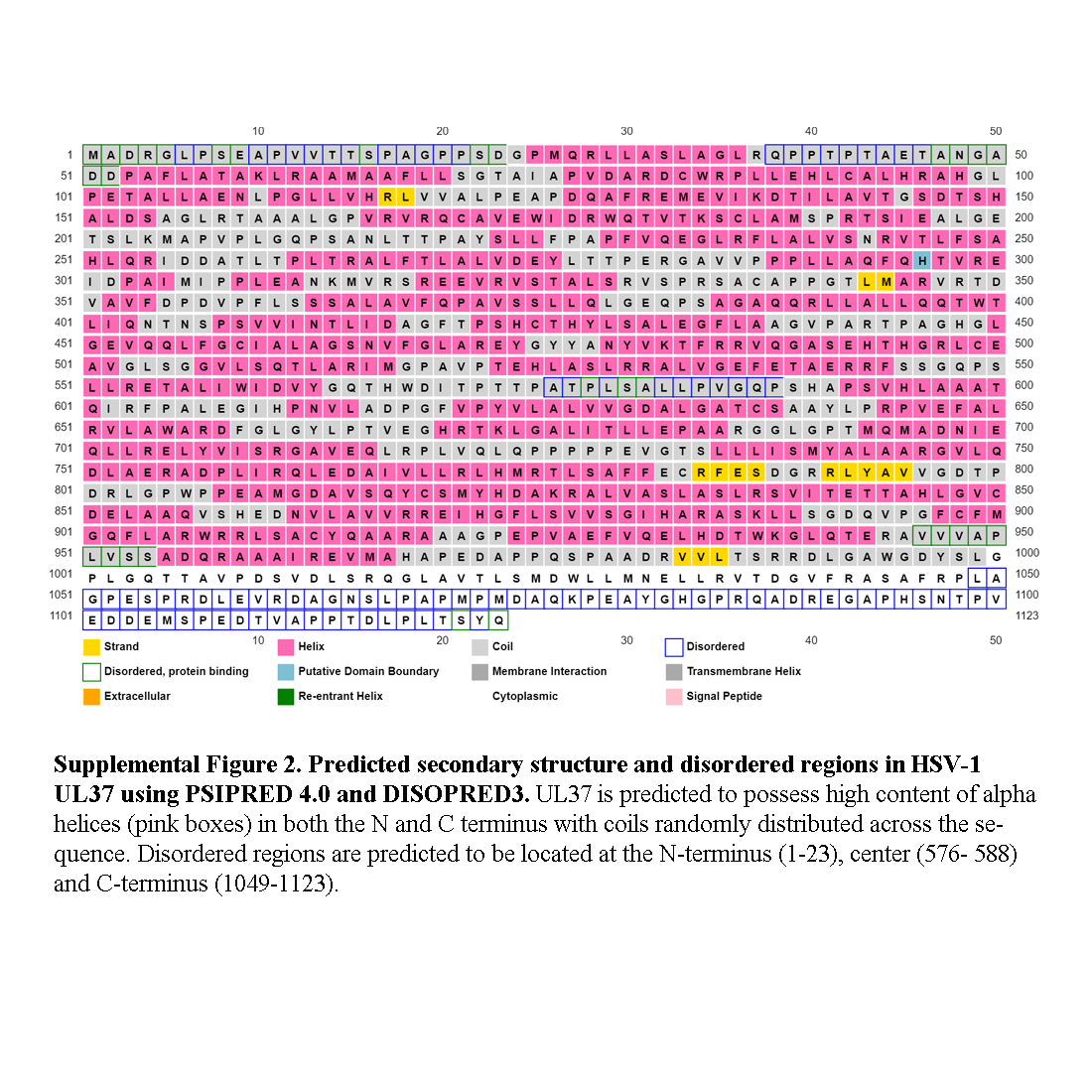

Supplement: Supplementary file 1 [file viruses-14-02189-s001.zip › Supplemental Figure S2.jpg]

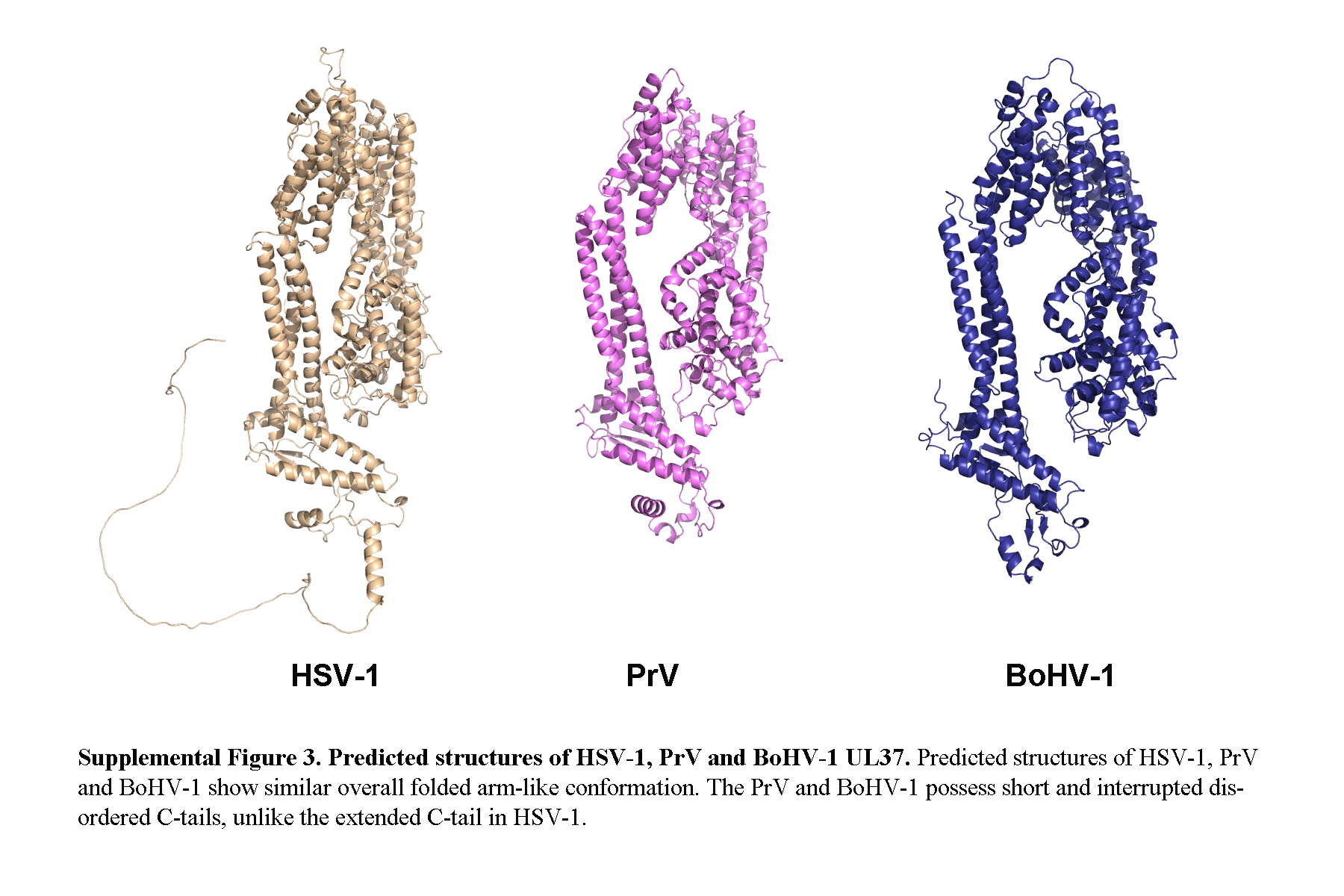

Supplement: Supplementary file 1 [file viruses-14-02189-s001.zip › Supplemental Figure S3.jpg]

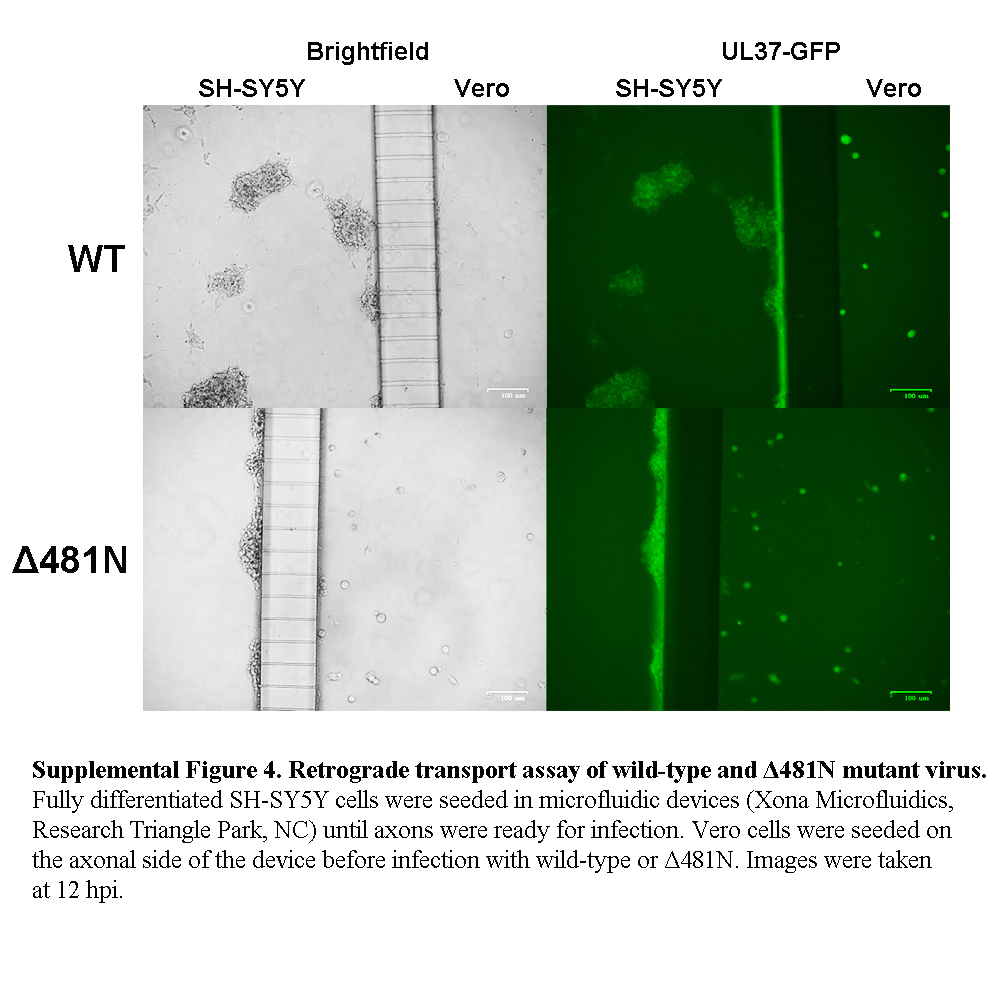

Supplement: Supplementary file 1 [file viruses-14-02189-s001.zip › Supplemental Figure S4.jpg]
